# An Unusual Presentation of Heat Rash: Bullous Miliaria in a Middle-Aged Woman

**DOI:** 10.7759/cureus.25323

**Published:** 2022-05-25

**Authors:** Fatema J Eshaqi, Zainab A Almaa, Fatima Samiey, Ameen Al Awadhi

**Affiliations:** 1 Dermatology, Salmaniya Medical Complex, Manama, BHR

**Keywords:** eccrine sweat glands, bullous, bullae, vesicles, miliaria crystallina, miliaria rubra, miliaria

## Abstract

Miliaria is a self-limiting cutaneous disease that may develop from skin exposure to humid climates, occlusion, or raised temperatures, forming groups of 1-3-mm-sized sweat-filled vesicles in the epidermis due to obstruction of the eccrine sweat duct. Here, we describe the first case of extensive bullous miliaria reported in a 47-year-old female with no comorbidities or significant medical history.

## Introduction

Miliaria crystallina occurs when sweat ducts in the most superficial layer of the epidermis, the stratum corneum, are obstructed. Miliaria rubra occurs when sweat ducts get blocked at the mid-epidermal level, and miliaria profunda is noted when the block occurs at the dermo-epidermal junction [1]. Risk factors include humid climates, occlusion, fever, and parasympathomimetic drugs, such as clonidine, neostigmine [2], and, rarely, doxorubicin [3], idarubicin, and oral tretinoin [4]. Miliaria is a condition that is typically diagnosed clinically, and laboratory investigations are usually not helpful. Bedside diagnostics such as dermoscopy and skin biopsy can help confirm the diagnosis in uncertain cases.

The vesicular lesions are typically 1-3 mm in diameter and self-resolve after removal of the triggering factors. Typically, patients experience superficial desquamation, but post-inflammatory hyper or hypopigmentation can also occur. The most serious complications include impaired thermoregulation and secondary bacterial infection.

To our knowledge, this is the first reported case of miliaria presenting with multiple bullous skin lesions admixed with smaller vesicles.

## Case presentation

A 47-year-old female, known to have glucose-6-phosphate dehydrogenase deficiency but otherwise healthy, presented to the emergency room with a five-day history of pruritic, elevated lesions all over the trunk, abdomen, and back that appeared one day after an episode of high-grade fever. Initially, the lesions appeared as grouped vesicles, which later became enlarged and/or coalesced, forming clear bullae with associated pruritus. The itch worsened during nighttime, impacting the patient’s ability to sleep. Along with the lesions on her abdomen, the patient presented with multiple erythematous maculopapular lesions on all four extremities. Apart from fever and itchiness, there were no other associated signs or symptoms.

Before presenting to the emergency department, the patient received topical and intravenous (IV) corticosteroids at the local health center; however, the lesions failed to subside.

**Figure 1:**
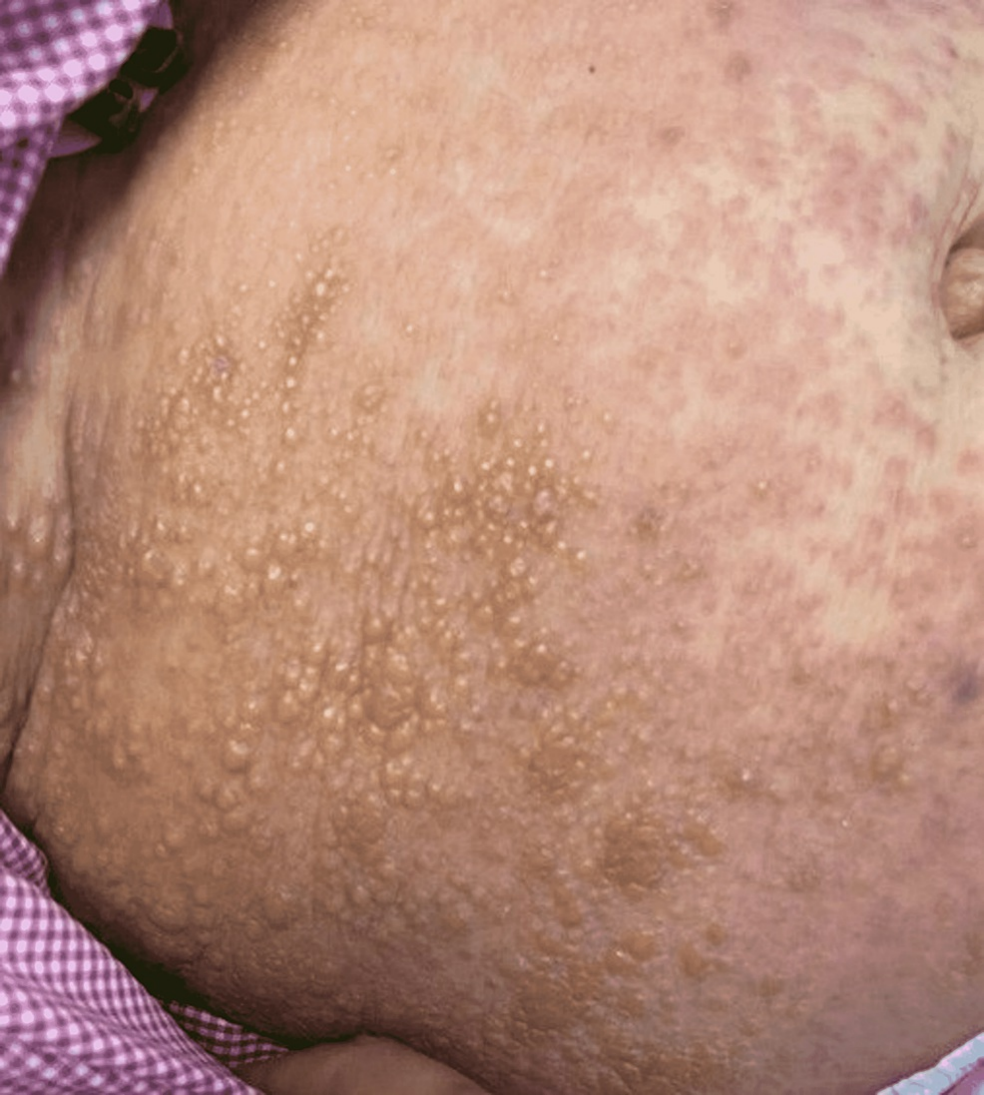
Multiple vesicles and bullae on an erythematous base on the right side of the abdomen.

**Figure 2:**
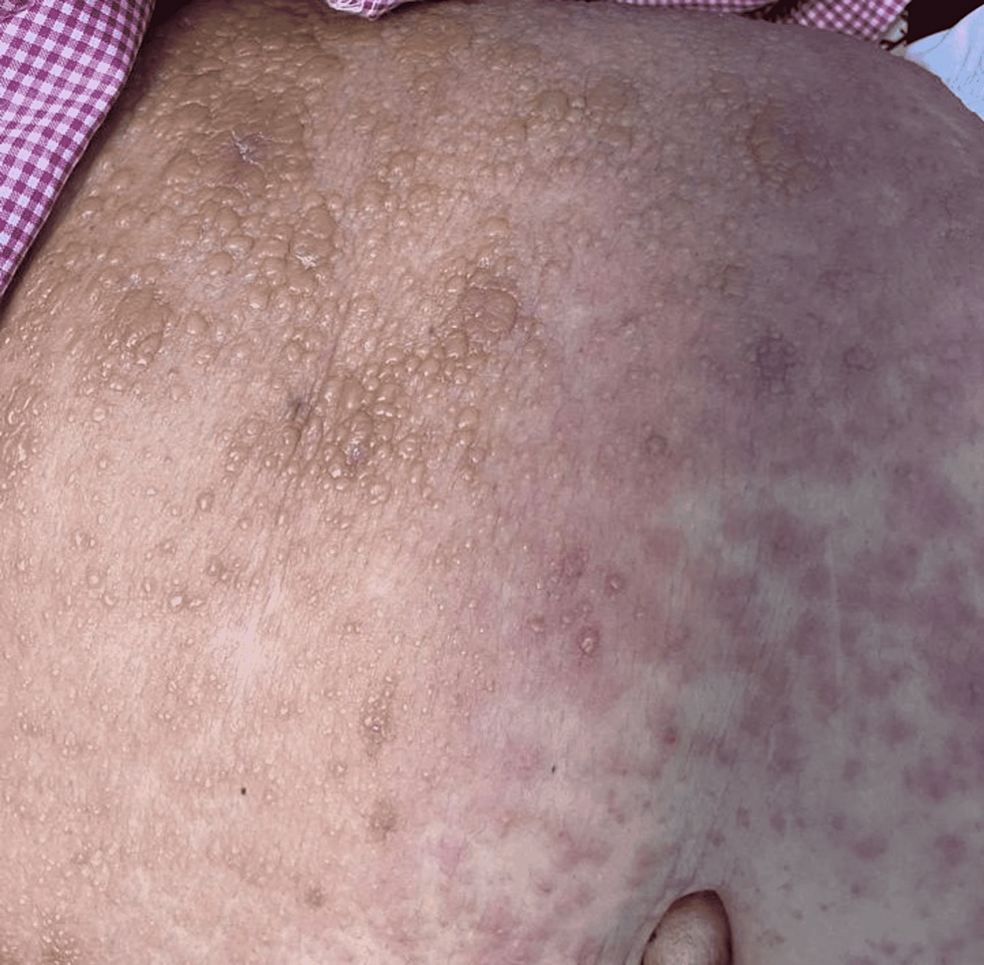
Multiple vesicles and bullae on an erythematous base on the left side of the abdomen.

**Figure 3:**
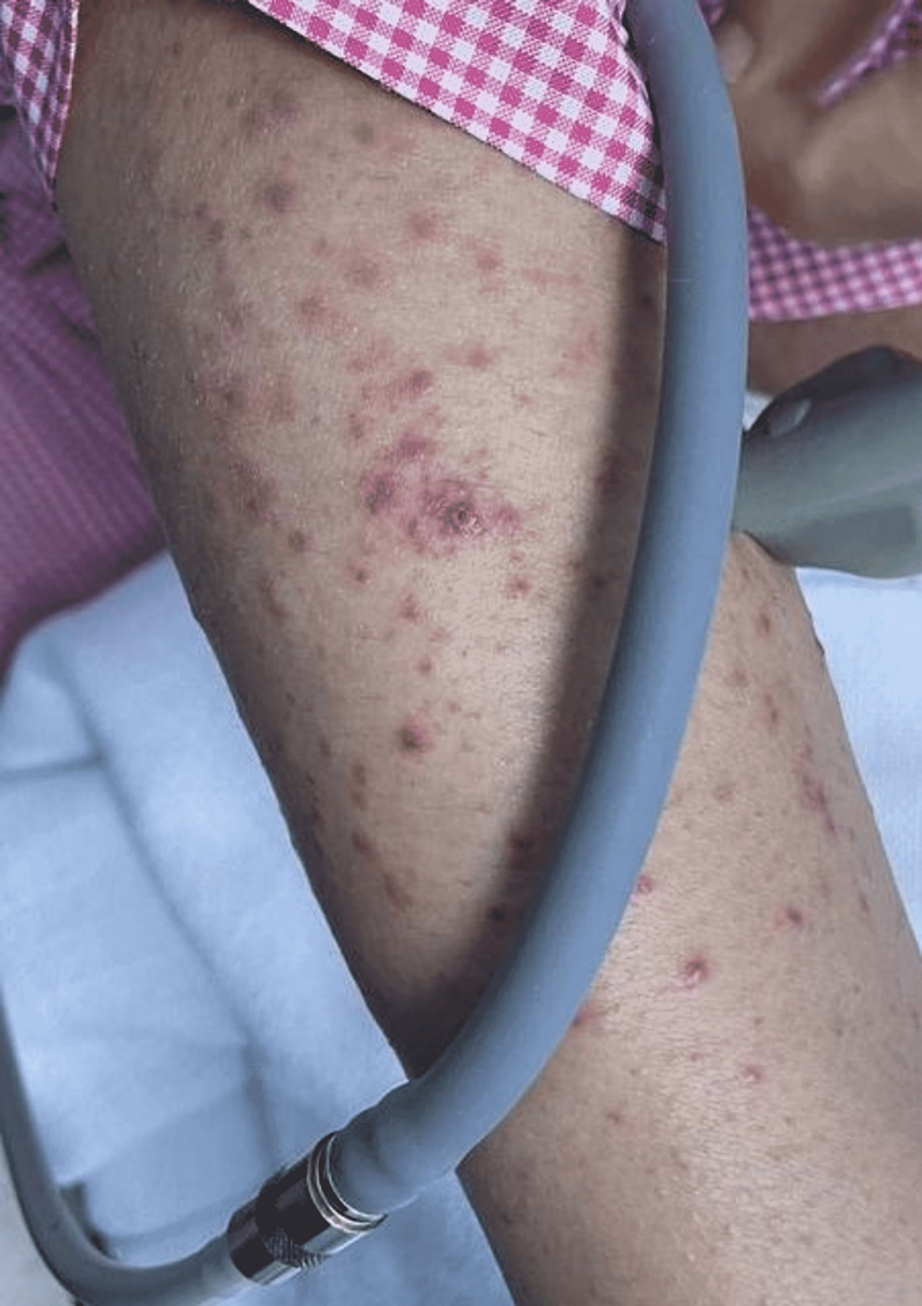
Multiple erythematous dusky papules and papulovesicles on the upper extremity.

**Figure 4:**
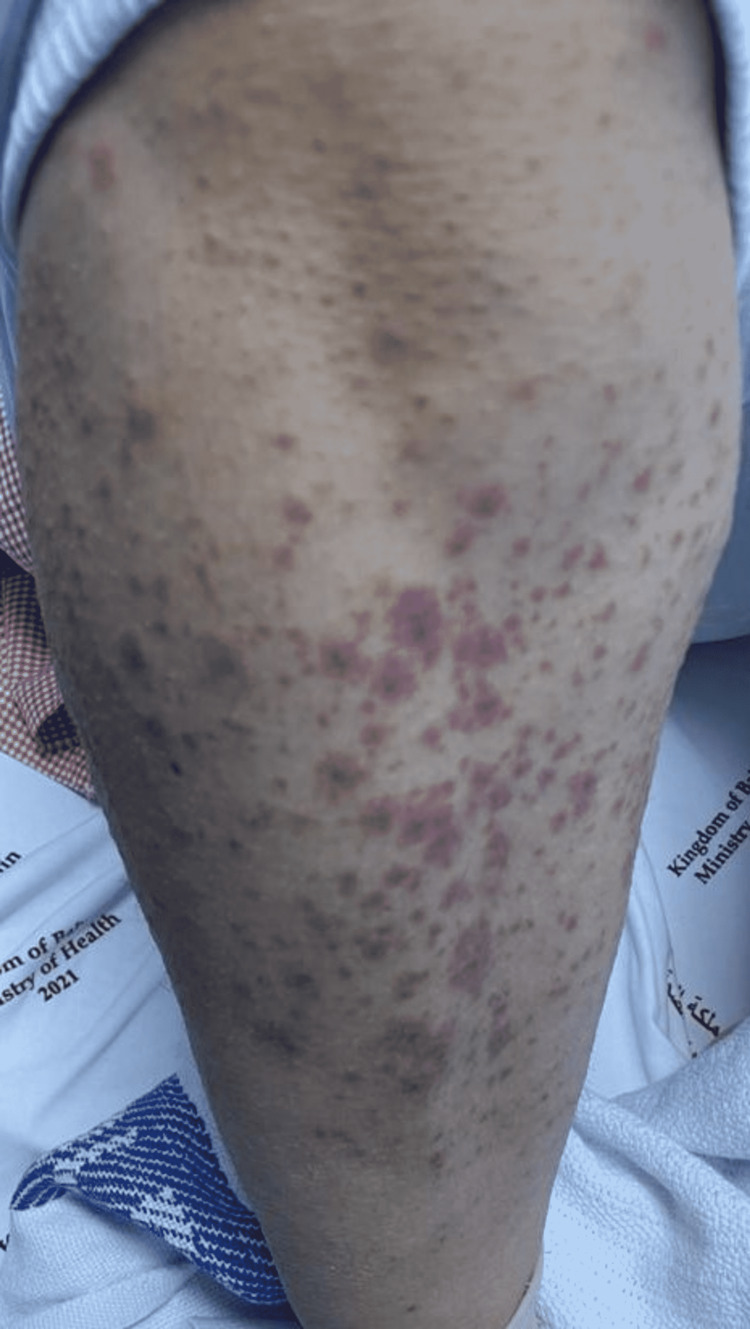
Multiple erythematous dusky papules and papulovesicles on the lower extremity.

Upon admission, a general physical examination and laboratory testing were done. On examination, the patient had stable vitals and clinically presented with multiple vesicles and bullae, on an erythematous base, mainly on the trunk, abdomen, and back (Figures 1, 2). When ruptured, these vesicles and bullae released clear fluid. Nikolsky’s sign was not appreciated, and a bedside Tzanck smear was unremarkable. In addition, the patient presented with multiple non-blanching erythematous macules and papules over the upper and lower extremities (Figures 3, 4). Cell blood count was notable for mild anemia. Additional tests including urinalysis, serum chemistries, and a vasculitic workup were non-revealing. Serological tests for herpes simplex, varicella, blood culture, and wound culture all were within normal limits.

The differential diagnosis at the time of admission included miliaria crystallina/rubra, bullous pemphigoid, viral exanthems, allergic dermatitis, disseminated herpes, varicella, and vasculitis.

**Figure 5:**
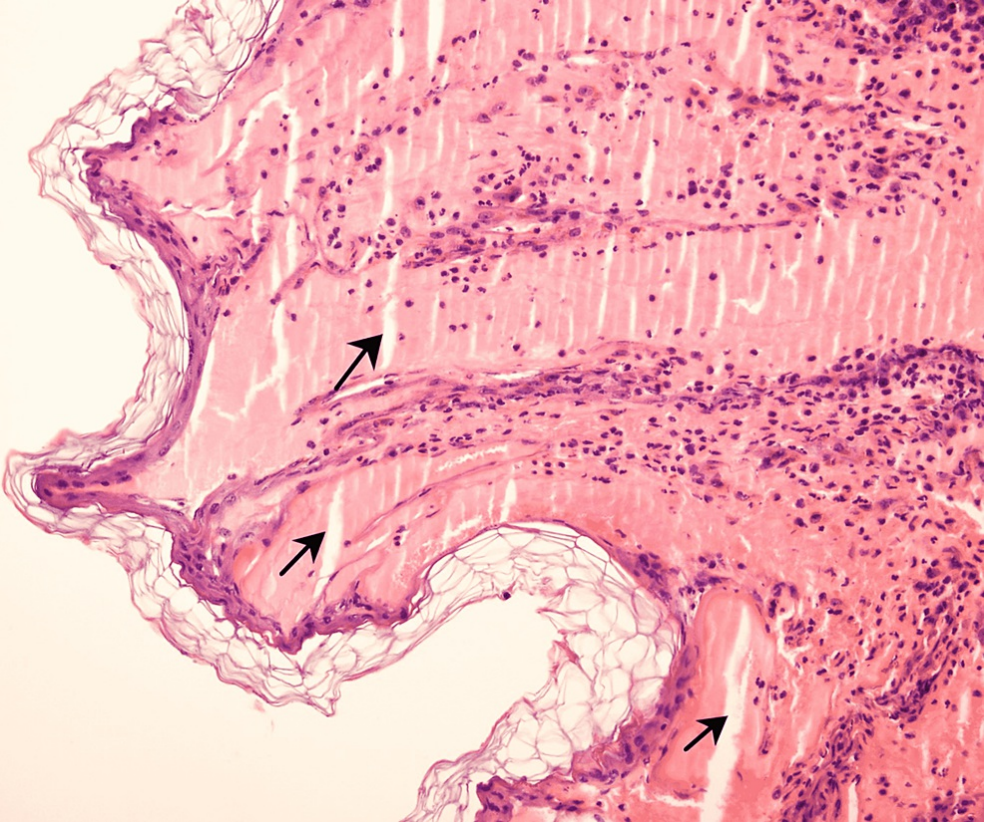
Intraepidermal edema separating the epidermal collagen fibers (H&E, 20×). H&E: hematoxylin and eosin

A 4 mm skin punch biopsy was obtained from the abdominal lesions for further characterization of the nature of the skin lesions and to assist in making the diagnosis. Hematoxylin and eosin staining of the epidermis revealed hyperkeratosis, focal parakeratosis, spongiosis, and exocytosis of lymphocytes and neutrophils. The dermis showed moderate perivascular lymphohistiocytic infiltrate without eosinophils or neutrophils. Histopathological evidence of vasculitis or viral infection was absent (Figure 5).

While waiting for the serology, laboratory, and pathology results, the patient was initially managed with IV acyclovir, topical steroids, cetirizine, paracetamol, cool compresses, and IV fluid support.

Over the next few days, while the patient was on empiric and supportive therapy, she did not develop any new lesions. She did have a single episode of hypotension, which was corrected by adjusting IV fluids.

The diagnosis of miliaria induced by fever was made based on the clinicopathological correlation and response to treatment; the type of miliaria was considered to be an overlap between crystallina and rubra. After allergic and infectious causes were ruled out, acyclovir was discontinued, and the patient was treated with antihistamines and topical steroids to control pruritus, as well as oral paracetamol with cold compresses to cool the body and reduce the fever.

A couple of days after admission, as the fever subsided, almost all the blisters receded; some ruptured and were replaced by serous crust, and the background erythema disappeared. The patient was subsequently fit for discharge with no medications. On the first follow-up visit a week later, all the previously persistent and crusted lesions had healed completely.

## Discussion

Although miliaria is common in newborns and infants due to the immaturity of sweat glands, it is also common in adults exposed to hot and humid environments. As detailed above, there are three main types, namely, crystallina, rubra, and profunda. Miliaria crystallina involves the eccrine ducts within the stratum corneum. The lesions are superficial, self-limiting, and can easily rupture. Miliaria rubra, the most common form, involves the eccrine ducts in the mid-epidermis, which usually shows an inflammatory response and tends to form larger vesicles with an erythematous base. These lesions are typically associated with pruritus and may form pustules. Miliaria profunda affects the eccrine ducts at the dermal-epidermal junction, forming non-pruritic, skin-colored papules, and is the rarest among all three types [1].

Our patient’s presentation with a sudden-onset pruritic fluid-filled vesicular and bullous eruption on her trunk, back, and abdomen was most consistent with a miliaria crystallina/rubra overlap.

What was unusual in this case presentation was that many of the lesions were bullae with a diameter larger than 1 cm. Miliaria usually is described as small vesicles and pustules that rarely reach sizes over 3 mm, making our case the first of its kind in describing bullous lesions in miliaria that were larger than 1 cm and reached up to 2 cm in some areas of the abdomen.

While allergic reactions, vasculitis, viral exanthems, and herpetic infections can have similar presentations as miliaria and had to be included in the differential diagnosis, history taking, physical examination, laboratory investigations, serology, and histopathology all helped in ruling out these entities and confirming the diagnosis of miliaria.

## Conclusions

Miliaria with multiple bullous lesions in a healthy individual is noteworthy, and our case is the first one describing this unusual presentation of a common skin disorder, which will help dermatologists as well as histopathologists in reaching a correct diagnosis when faced with a similar presentation. Through this article, we wish to emphasize the importance of keeping a broad differential diagnosis in approaching such unsuspected cases.

## References

[1] Bolognia JL, Schaffer JV, Cerroni L (2017). Diseases of the eccrine and apocrine sweat glands. Dermatology.

[2] Haas N, Martens F, Henz BM (2004). Miliaria crystallina in an intensive care setting. Clin Exp Dermatol.

[3] Seghers AC, Tey HL, Tee SI, Cao T, Chong WS (2018). Pegylated liposomal doxorubicin-induced miliaria crystallina and lichenoid follicular eruption. Indian J Dermatol Venereol Leprol.

[4] Valenzuela-Ubiña S, Villegas-Romero I, Jiménez-Gallo D, Arjona-Aguilera C, Linares-Barrios M (2021). Miliaria crystallina induced by idarubicin and all-trans-retinoic acid: two case reports. Australas J Dermatol.

